# Brief Report: Hydroxychloroquine does not induce hemolytic anemia or organ damage in a “humanized” G6PD A- mouse model

**DOI:** 10.1371/journal.pone.0240266

**Published:** 2020-10-02

**Authors:** Benjamin E. Zuchelkowski, Ling Wang, Sebastien Gingras, Qinzi Xu, Minying Yang, Darrell Triulzi, Grier P. Page, Victor R. Gordeuk, Daniel B. Kim-Shapiro, Janet S. Lee, Mark T. Gladwin

**Affiliations:** 1 Department of Medicine, Division of Pulmonary, Allergy and Critical Care Medicine, University of Pittsburgh School of Medicine, Pittsburgh, PA, United States of America; 2 Pittsburgh Heart, Lung, Blood and Vascular Medicine Institute, Pittsburgh, PA, United States of America; 3 Department of Immunology, University of Pittsburgh School of Medicine, Pittsburgh, PA, United States of America; 4 Department of Pathology, Division of Transfusion Medicine, University of Pittsburgh School of Medicine, Pittsburgh, PA, United States of America; 5 RTI International, Research Triangle Park, Durham, NC, United States of America; 6 Division of Hematology and Oncology, University of Illinois at Chicago School of Medicine, Chicago, IL, United States of America; 7 Department of Physics, Wake Forest University, Winston-Salem, NC, United States of America; Yale University School of Medicine, UNITED STATES

## Abstract

**Background:**

Hydroxychloroquine (HCQ) is widely used in the treatment of malaria, rheumatologic disease such as lupus, and most recently, COVID-19. These uses raise concerns about its safe use in the setting of glucose-6-phosphate dehydrogenase (G6PD) deficiency, especially as 11% of African American men carry the G6PD A- variant. However, limited data exist regarding the safety of HCQ in this population.

**Study design and methods:**

Recently, we created a novel “humanized” mouse model containing the G6PD deficiency A- variant (Val68Met) using CRISPR technology. We tested the effects of high-dose HCQ administration over 5 days on hemolysis in our novel G6PD A- mice. In addition to standard hematologic parameters including plasma hemoglobin, erythrocyte methemoglobin, and reticulocytes, hepatic and renal function were assessed after HCQ.

**Results:**

Residual erythrocyte G6PD activity in G6PD A- mice was ~6% compared to wild-type (WT) littermates. Importantly, we found no evidence of clinically significant intravascular hemolysis, methemoglobinemia, or organ damage in response to high-dose HCQ.

**Conclusions:**

Though the effects of high doses over prolonged periods was not assessed, this study provides early, novel safety data of the use of HCQ in the setting of G6PD deficiency secondary to G6PD A-. In addition to novel safety data for HCQ, to our knowledge, we are the first to present the creation of a “humanized” murine model of G6PD deficiency.

## Introduction

Hydroxychloroquine (HCQ) is a mainstay of therapy for malaria and rheumatologic diseases. Recently, it has been used as a possible therapeutic in COVID-19. These uses have presented concerns about possible toxicity of the medication if used in patients with glucose-6-phosphate dehydrogenase (G6PD) deficiency, related to reports in the 1950s of “primaquine sensitivity” and hemolytic anemia related to underlying G6PD deficiency [1]. Because 11% of African American males carry the A- variant of G6PD associated with low enzyme activity, and there is a paucity of high-quality safety evidence for the use of HCQ in patients with G6PD deficiency, further study is needed to adequately assess the risk of hemolytic anemia in this population. G6PD A- individuals do not undergo chronic hemolysis, but they are at risk for hemolytic episodes when exposed to oxidant stress.

Some studies suggest that HCQ can be safely administered in the setting of G6PD deficiency [2, 3]. In a retrospective chart review of 275 rheumatology patients taking HCQ chronically, 11 African American patients with G6PD deficiency were evaluated through 700 months of therapy, without evident complications [2]. Additionally, chloroquine did not induce hemolysis in a heterozygote knock-out murine model of G6PD deficiency with 14% residual enzyme activity [3]; however, the model’s specific hypomorphic mutation is not present in humans.

In order to directly test the safety of HCQ in the setting of G6PD A-, we developed a novel, “humanized” murine model of this variant (*G6PD* V68M) using CRISPR-Cas9 technology and treated the mice with high dose HCQ over 5 days by intraperitoneal injection. To our knowledge, this is the first report of HCQ exposure in a humanized murine model of G6PD A-.

## Materials and methods

In the murine model, the desired single nucleotide polymorphism (rs1050828, Val68Met), valine 68 was substituted to methionine using CRISPR/Cas9 technology [4, 5] using an oligonucleotide template for homology directed repair (HDR) containing the homologous human DNA sequence [6]. The 376G allele (126 Asn → Asp) of the human G6PD A- variant is absent from this model because it does not produce significant disruptions in enzyme activity that would otherwise alter the hemolytic phenotype [7]. A manuscript detailing the generation and characterization of the G6PD-V68M is in preparation. Founder mice and N1 offspring were confirmed through sequence analysis and a stable colony was obtained through strategic back-crossing to wild-type mice. Erythrocyte G6PD activity was measured using fluorometric-based method (Cayman Chemicals, Ann Arbor, MI).

We evaluated the *in vivo* hemolytic propensity of G6PD A- mouse RBCs to HCQ sulfate (Cayman Chemicals). Wild-type (WT) littermate male or G6PD A- male mice (n = 10/group) at 13-weeks were administered 60 mg/kg HCQ by intraperitoneal injection every 24 hrs for 5 days. This dose is higher than the 50 mg/kg dose equivalent to a human dose for anti-malarial prophylaxis [8]. Murine tail vein blood (70 μL) was sampled from each mouse on experimental day 1 prior to receiving drug. On day 6, whole blood was collected via the inferior vena cava immediately following euthanasia using heparin as an anti-coagulant (Sigma, St. Louis, MO). Reticulocytes were enumerated by flow cytometry. Complete blood counts were obtained by a commercial blood counter (Hemavet 950FS, Drew Scientific, Miami FL). Whole blood was centrifuged at 1800 *g* for 10 min at 18°C. Plasma and RBC pellets were stored separately at -80°C until later use. The study was approved by the University of Pittsburgh Institutional Animal Care and Use Committee (IACUC).

Intraerythrocytic hemoglobin, methemoglobin, and extracellular plasma hemoglobin concentrations were determined by spectroscopy using a least-squares deconvolution with reference spectra [9]. For extracellular plasma hemoglobin we report concentrations in terms of heme concentration; note [hemoglobin]_plasma_ = [heme]_plasma_/4. Plasma haptoglobin levels were determined by enzyme-linked immunosorbent assays (ELISA) according to manufacturer’s instructions (Abcam, Cambridge, UK). A complete metabolic panel was performed on plasma (IDEXX Bio Analytics, North Grafton, MA). Data was presented as means ± SEM. GraphPad Prism 7.03 was used for all statistical analyses and the details on statistical analyses are indicated in the legends.

## Results

**Fig 1:**
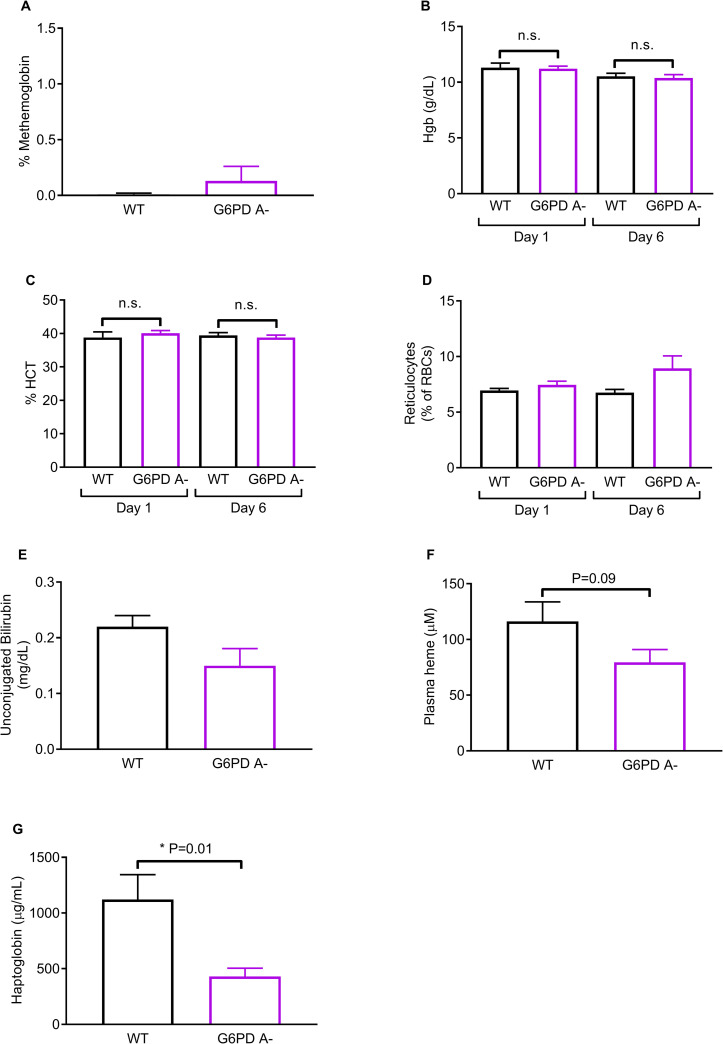
HCQ does not induce significant *in vivo* hemolysis in G6PD A- mice with low residual Red Blood Cell (RBC) G6PD enzyme activity. WT and G6PD A- male mice (n = 10/group) were administered 60 mg/kg HCQ i.p. daily for 5 days, then whole blood collected via inferior vena cava using heparin as an anti-coagulant following anesthesia. **A**, Quantitative spectral analysis of RBC Methemoglobin (%) **B-C**, CBC on whole blood by Hemavet performed on Day 1 and Day 6, **B,** hemoglobin (g/dL), **C,** hematocrit (%); **D**, Reticulocytes were enumerated by flow cytometry on Day 1 pre-HCQ and on Day 6 post-HCQ from 5-μL whole blood and expressed as percentage of RBCs. **E,** Unconjugated bilirubin (mg/dL) determined by commercial complete metabolic panel; **F**, Plasma hemoglobin reported as heme concentration (μM) determined by quantitative spectral analysis [9] **G**, Plasma haptoglobin (μg/mL) determined by ELISA; **A-G**, bars = mean ± SEM, statistical significance was determined using repeated measures ANOVA with Bonferroni’s multiple comparisons for Hgb and % HCT, Kruskall-Wallis test with Dunn’s multiple comparisons for % Reticulocytes because assumptions supporting parametric analysis were not met, or unpaired Student’s t-test for two-group comparisons except where * denotes Mann-Whitney-U was used for haptoglobin because assumptions supporting parametric analysis were not met; in **D** and **G,** assumptions of normality and equal variance were not met, bold text emphasizes significance of findings at P<0.05, n.s. = non-significant.

We found that high dose HCQ did not induce significant hemolysis or organ damage in G6PD A- mice. Clinical characteristics of WT and G6PD A- mice are provided in Table 1. Residual RBC G6PD enzyme activities in G6PD A- mice are 6.5 ± 0.083% of the levels in WT. We did not observe significant differences in relevant hematologic parameters that would otherwise indicate a hemolytic anemia, including RBC counts, hemoglobin and hematocrit (Fig 1B and 1C), mean corpuscular volume (MCV), or mean corpuscular hemoglobin (MCH) (Table 1). There was a slight drop in hemoglobin after HCQ treatment, though comparisons of absolute values were not significant (Table 1) and the interaction term (time*genotype) was not significant (p = 0.88), indicating the effect is not genotype-dependent. Additionally, we found that the change in hemoglobin levels was not significantly different (Table 1), indicating the time-dependent drop in hemoglobin is likely due to phlebotomy or measurement variation. There were no significant differences in RBC methemoglobin levels post-HCQ. Reticulocyte percentage of RBCs were not significantly different in either group at baseline (6.95 ± 0.18% in WT vs 7.45 ± 0.33% in G6PD A-, p > 0.99) or following HCQ exposure (6.76 ± 0.29% in WT vs 8.93 ± 1.12% in G6PD A-, p = 0.61; Fig 1D). There were no significant differences in unconjugated bilirubin (0.22 ± 0.02 mg/dL in WT vs 0.15 ± 0.031 in G6PD A-, p = 0.07; Fig 1E, Table 1). There were no significant differences in extracellular plasma hemoglobin (116.20 ± 17.56 μM in WT vs 79.45 ± 11.48 μM in G6PD A-, p = 0.09, Fig 1F) post-HCQ. Plasma haptoglobin was significantly lower in G6PD A- compared to WT (430.6 ± 73.88 μg/mL in G6PD A- vs 1122 ± 222.1 μg/mL in WT, p = 0.01; Fig 1G). There were no differences in aspartate aminotransferase (AST), alanine aminotransferase (ALT), alkaline phosphatase (ALP), creatinine kinase (CK), albumin, blood urea nitrogen (BUN), or creatinine between WT and G6PD A- groups, suggesting no enzymatic and laboratory evidence of hemolysis, or hepatic and kidney injury (Table 1). Finally, G6PD A- mice had higher mean glucose levels compared to WT littermates (Table 1), consistent with clinical studies demonstrating increased incidence of diabetes mellitus and/or impaired fasting glucose in patients with G6PD deficiency [10].

**Table 1:** Selected hematologic and metabolic parameters of WT and G6PD A- mice.

*Hematologic Parameters*		*WT*		*G6PD A-*	*P-value*
	n	mean ± SEM	n	mean ± SEM	
RBC (1x10^6^/μL)					
Baseline	10	8.40 ± 0.28	10	8.38 ± 0.15	>0.99
Day 6	10	8.26 ± 0.19	10	8.03 ± 0.16	0.88
Absolute change after 6 days	--	-0.15 ± 0.27	--	-0.35 ± 0.15	0.52
% Change	--	-0.98 ± 3.35	--	-4.07 ± 1.73	
Hgb (g/dL)					
Baseline	10	11.31 ± 0.41	10	11.22 ± 0.22	>0.99
Day 6	10	10.53 ± 0.28	10	10.38 ± 0.30	>0.99
Absolute change after 6 days	--	-0.78 ± 0.28	--	-0.84 ± 0.26	0.88
% Change	--	-6.34 ± 2.40	--	-7.43 ± 2.30	
HCT (%)					
Baseline	10	38.82 ± 1.66	10	40.09 ± 0.80	0.81
Day 6	10	39.45 ± 0.82	10	38.82 ± 0.70	>0.99
Absolute change after 6 days	--	0.63 ± 1.70	--	-1.27 ± 0.69	0.31
% Change	--	3.34 ± 4.98	--	-3.01 ± 1.68	
MCV (fL)					
Baseline	10	47.41 ± 0.17	10	47.84 ± 0.34	0.55
Day 6	10	47.81 ± 0.18	10	48.38 ± 0.35	0.30
Absolute change after 6 days	--	0.4 ± 0.18	--	0.54 ± 0.21	0.62
% Change	--	0.85 ± 0.38	--	1.14 ± 0.45	
MCH (pg)					
Baseline	10	13.45 ± 0.089	10	13.40 ± 0.12	>0.99
Day 6	10	12.77 ± 0.15	10	12.91 ± 0.18	0.98
Absolute change after 6 days	--	-0.68 ± 0.47	--	-0.49 ± 0.66	0.47
% Change	--	-5.04 ± 3.48	--	-3.60 ± 4.91	
G6PD Activity (% of WT)	10	--	10	6.5 ± 0.083	
*Metabolic Parameters (Day 6)*					
AST (U/L)	10	34.70 ± 1.46	10	36.70 ± 2.87	0.54
ALT (U/L)	10	12.1 ± 1.04	10	11.6 ± 1.08	0.75
ALP (U/L)	10	595.6 ± 252.80	10	398 ± 13.82	0.47*
Total Bilirubin (mg/dL)	10	0.23 ± 0.015	10	0.14 ± 0.037	0.07*
Conjugated (mg/dL)	--	0.01 ± 0.010	--	0.04 ± 0.017	0.27*
Unconjugated (mg/dL)	--	0.22 ± 0.02	--	0.15 ± 0.031	0.12*
Creatine Kinase (U/L)	10	34.60 ± 8.11	10	71.60 ± 34.8	0.96*
Total Protein (g/dL)	10	4.32 ± 0.051	10	4.29 ± 0.048	0.67
Albumin (g/dL)	10	2.14 ± 0.053	10	2.10 ± 0.046	0.58
Glucose (mg/dL)	10	138.10 ± 12.11	10	176.20 ± 8.50	**0.02**
BUN (mg/dL)	10	262.2 ± 9.07	10	244 ± 13.99	0.29
Creatinine (mg/dL)	10	0.012 ± 0.002	10	0.01 ± 0.0	0.33
Calcium (mg/dL)	10	7.57 ± 0.14	10	7.58 ± 0.12	0.96
Phosphorous (mg/dL)	10	4.06 ± 0.24	10	4.51 ± 0.16	0.13

Hematologic parameters were measured from heparinized whole blood on day 1 (baseline) and day 6 (post-HCQ) by commercial blood counter (Hemavet). Metabolic parameters were measured in a commercial lab (IDEXX Bio Analytics) from plasma collected on day 6. Statistical significance was determined using repeated measures ANOVA with Bonferroni’s multiple comparisons for absolute hematologic values, or Student’s t-test for single or change comparisons except where * denotes Mann-Whitney-U was used because assumptions supporting parametric analysis were not met; bold text emphasizes significance of findings at P<0.05.

*ALP*, *Total Bilirubin*, *Conjugated Bilirubin*, *Unconjugated Bilirubin*, *CK*: Parametric assumptions of normality and equal variance not met

*Definition of abbreviations*: RBC = red blood cell; HCT = hematocrit; MCV = mean corpuscular volume; MCH = mean corpuscular hemoglobin; G6PD = glucose-6-phosphate dehydrogenase; AST = aspartate aminotransferase; ALT = alanine aminotransferase; ALP = alkaline phosphatase; BUN = blood urea nitrogen.

## Discussion

Overall, our study suggests that short-course high doses of HCQ do not induce methemoglobinemia or clinically significant hemolytic anemia or organ damage in our murine model of G6PD deficiency. While we cannot exclude low level hemolysis lowering haptoglobin concentrations, this was insufficient to produce methemoglobinemia, lower hemoglobin levels, hematocrit and reticulocyte percentage, or increase indirect bilirubin and AST. These findings are consistent with prior retrospective clinical data in limited numbers of G6PD deficient patients [2]. It is also noteworthy that residual G6PD erythrocyte enzyme activity in our model is lower than in previous studies [3]. Though the residual enzyme activity in this model could be strictly classified as a Class IV mutation according to WHO classification, the phenotype is more consistent with a Class III mutation. Of note, this G6PD A- mouse model is ideal for future studies in transfusion medicine.

Although this report provides further novel safety data for HCQ usage in G6PD A- mice, there are several limitations. The effect of long-term HCQ in our G6PD A- mice was not assessed. Further, the combined effect of systemic infection and HCQ, such as in the setting of treatment for severe COVID-19, on hemolytic propensity was not assessed. LPS has been shown to induce hemolysis via direct membrane interaction [11]. Therefore, questions remain regarding the nature of this combined effect on hemolytic propensity in G6PD A- and clinicians should still exercise caution when considering HCQ in this setting.

## Supporting information

S1 Raw data Raw experimental data. (XLSX)

## References

[1] Luzzatto L, Nannelli C, Notaro R. Glucose-6-Phosphate Dehydrogenase Deficiency. Hematol Oncol Clin North Am. 2016 4;30(2):373–393. 10.1016/j.hoc.2015.11.006 27040960

[2] Mohammad S, Clowse MEB, Eudy AM, Criscione-Schreiber LG. Examination of Hydroxychloroquine Use and Hemolytic Anemia in G6PDH-Deficient Patients. Arthritis Care Res (Hoboken). 2018 2 9;70(3):481–485. 28556555 10.1002/acr.23296

[3] Zhang P, Gao X, Ishida H, Amnuaysirikul J, Weina PJ, Grogl M, et al An in vivo drug screening model using glucose-6-phosphate dehydrogenase deficient mice to predict the hemolytic toxicity of 8-aminoquinolines. Am J Trop Med Hyg. 2013 6;88(6):1138–1145. 10.4269/ajtmh.12-0682 23530079 PMC3752814

[4] Martinez J, Malireddi RKS, Lu Q, Cunha LD, Pelletier S, Gingras S, et al Molecular characterization of LC3-associated phagocytosis reveals distinct roles for Rubicon, NOX2 and autophagy proteins. Nat Cell Biol. 2015 7;17(7):893–906. 10.1038/ncb3192 26098576 PMC4612372

[5] Wang H, Yang H, Shivalila CS, Dawlaty MM, Cheng AW, Zhang F, et al One-step generation of mice carrying mutations in multiple genes by CRISPR/Cas-mediated genome engineering. Cell. 2013 5 9;153(4):910–918. 10.1016/j.cell.2013.04.025 23643243 PMC3969854

[6] Wang L, Zuchelkowski B, Sinchar D, Yang M, Gingras S, Kanias T, et al Evaluation of the Functional Effects of an African American Glucose-6-Phosphate Dehydrogenase (G6PD) Polymorphism (Val68Met) on RBC Hemolytic Propensity and Post-Transfusion Recovery in a Humanized Mouse Model. Blood. 2019 11 13;134(Supplement_1):102–102.

[7] Town M, Bautista JM, Mason PJ, Luzzatto L. Both mutations in G6PD A- are necessary to produce the G6PD deficient phenotype. Hum Mol Genet. 1992 6;1(3):171–174. 10.1093/hmg/1.3.171 1303173

[8] Masson J-D, Blanchet B, Periou B, Authier F-J, Mograbi B, Gherardi RK, et al Long term pharmacological perturbation of autophagy in mice: are HCQ injections a relevant choice? Biomedicines. 2020 3 1;8(3). 10.3390/biomedicines8030047 PMC7148514 32121613

[9] Donadee C, Raat NJH, Kanias T, Tejero J, Lee JS, Kelley EE, et al Nitric oxide scavenging by red blood cell microparticles and cell-free hemoglobin as a mechanism for the red cell storage lesion. Circulation. 2011 7 26;124(4):465–476. 10.1161/CIRCULATIONAHA.110.008698 21747051 PMC3891836

[10] Santana MS, Monteiro WM, Costa MRF, Sampaio VS, Brito MAM, Lacerda MVG, et al High frequency of diabetes and impaired fasting glucose in patients with glucose-6-phosphate dehydrogenase deficiency in the Western brazilian Amazon. Am J Trop Med Hyg. 2014 7;91(1):74–76. 10.4269/ajtmh.13-0032 24865682 PMC4080572

[11] Brauckmann S, Effenberger-Neidnicht K, de Groot H, Nagel M, Mayer C, Peters J, et al Lipopolysaccharide-induced hemolysis: Evidence for direct membrane interactions. Sci Rep. 2016 10 19;6:35508 10.1038/srep35508 27759044 PMC5069489

